# Oblique Lumbar Interbody Fusion with Stand-Alone Cages for the Treatment of Degenerative Lumbar Spondylolisthesis: A Retrospective Study with 1-Year Follow-Up

**DOI:** 10.1155/2020/9016219

**Published:** 2020-01-11

**Authors:** Yachong Huo, Dalong Yang, Lei Ma, Haidong Wang, Wenyuan Ding, Sidong Yang

**Affiliations:** Department of Spinal Surgery, The Third Hospital of Hebei Medical University, Shijiazhuang, Hebei 050051, China

## Abstract

Patients with degenerative lumbar spondylolisthesis (DLS) often suffer from years of low back pain (LBP) due to instability of the lumbar spine and the reduction of disc height. Since January 2016, we have performed oblique lateral interbody fusion (OLIF) on 154 patients. Among these, 56 patients who suffered from DLS underwent OLIF with stand-alone cages. Forty-two patients with a follow-up time that exceeded 1-year were enrolled for this study. The forty-two patients were followed up for at least one year. Operation segments ranged from L3-4 to L4-5. All the patients were with 1-level fusion. The mean postoperative ventral-disc height and dorsal-disc height increased significantly compared with preoperative (*P* < 0.05). A significant postoperative increase was also observed in the mean operative segmental lordotic angle and the whole lumbar lordotic angle (*P* < 0.05). Compared with preoperative, the postoperative VAS significantly decreased with no significant increase in the VAS in the last follow-up. The LBP was significantly relieved. The mean postoperative VAS of LBP decreased significantly compared with the preoperative ((1.6 ± 0.8) vs. (7.8 ± 0.8)). Postoperative complications included psoas major abscess and intervertebral space infection (1/56). Except for one patient whose cage subsided during the last follow-up, the other patients had good cage position. The one whose cage collapsed complained no symptoms including LBP. OLIF with stand-alone cages should be considered as a safe and effective option which can effectively alleviate LBP for the treatment of DLS.

## 1. Introduction

Lumbar interbody fusion has been widely used for the treatment of patients with degenerative lumbar spondylolisthesis (DLS) who have failed with nonoperation therapy. DLS mainly leads to low back pain (LBP), whereas posterior or combined anterior-posterior approaches may cause postoperative LBP due to the dissection of paravertebral muscles. Anterior or anterolateral approach surgeries for lumbar interbody fusion have been increasingly popular due to the protection of paravertebral muscles. Compared with the anterior lumbar interbody fusion (ALIF), oblique lumbar interbody fusion (OLIF) is an extraperitoneal approach with lower incidence of abdominal complications, vascular injury, and reverse ejaculation [1, 2]. Patients with DLS often suffer from years of LBP due to instability of the lumbar spine and the reduction of disc height.

Recently, studies have proved that stand-alone cages in ALIF can effectively restore the disc height and stabilize the spine promoting fusion [1]. But peritoneal injury and intestinal complications are inevitable. We recently attempted using OLIF with stand-alone cages for the treatment of DLS to provide immediate stability. The purpose of our study was to assess the feasibility and clinical outcomes of patients with DLS after this technique.

## 2. Materials and Methods

### 2.1. Study Population

Since January 2016, we have performed the OLIF procedure on 154 patients. Among these, 56 patients who suffered from DLS underwent OLIF with stand-alone cages. All of the main complaint was ineffective conservative therapy of LBP. But there existed no isthmic fissure slip on all the cases. Forty-two patients with a follow-up time that exceeded 1-year were enrolled for this study (Table 1).

### 2.2. Surgical Procedures

**Figure 1:**
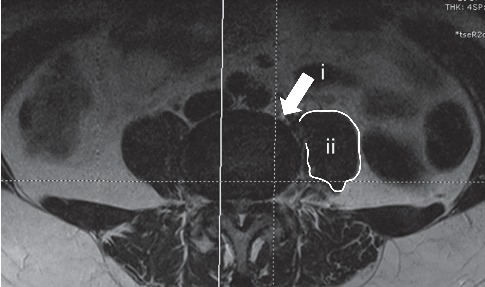
OLIF technology approach. From the space between the psoas muscle (ii) and blood vessel to the target intervertebral space (i).

Under general anesthesia, OLIF was performed with the patient in the left lateral position. Conventional OLIF, including marking incision, blunt dissection of muscle, and application of retractor, was applied. After discectomy, the suitable size stand-alone cage was inserted to the discectomy-disc field (Figure 1).

### 2.3. Analysis of Radiological Parameters

**Figure 2:**
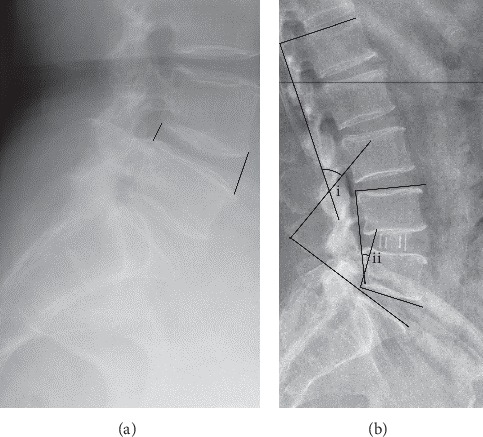
Measurement method. (a) Measurement diagram of intervertebral disc height; (b) the whole lumbar lordotic angle (i) and the operative segmental lordotic angle (ii).

Radiological parameters that we analyzed included ventral-disc height (VDH), dorsal-disc height (DDH), operative segmental lordotic angle, and the whole lumbar lordotic angle (LL) by comparing the preoperative and postoperative sagittal X-Ray. All the data were measured by two researchers and averaged. Another expert was asked to evaluate the discordant data to ensure the accuracy. Ventral-disc height was measured as the distance between anterior-inferior intersection of the lower vertebral body and anterior-superior intersection of the upper vertebral body. Dorsal-disc height was measured as the distance between posterior-inferior intersection of the lower vertebral body and posterior-superior intersection of the upper vertebral body. The operative segmental lordotic angle was measured as the angle between the upper endplate of the upper vertebral body and the lower endplate of the lower vertebral body. The whole lumbar lordotic angle was measured as the angle between the upper endplate of L1 and the upper endplate of S1 (Figure 2).

### 2.4. Analysis of Clinical Parameters

Clinical outcome parameters including the Oswestry Disability Index (ODI) and visual analog scale (VAS) were investigated. Operation time, estimated blood loss, and procedure-related complications were recorded. Each patient was followed up prospectively with preoperative and postoperative evaluations (2 weeks, 3 months, and last follow-up).

### 2.5. Statistical Analysis

Statistical analysis was performed by using SPSS version 22.0 for Windows. Radiological and clinical parameters were all compared by using the independent-sample *T* test. *P* < 0.05 was considered statistically significant.

## 3. Results

**Figure 3:**
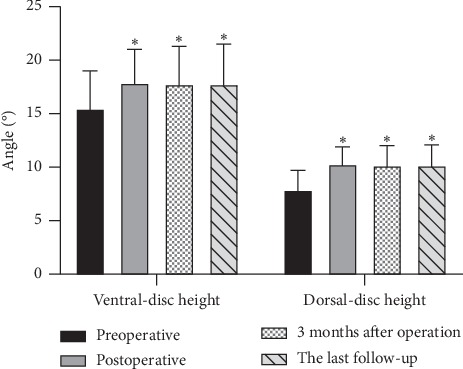
The comparisons of intervertebral disc height. Compared with preoperative, the height of ventral and dorsal intervertebral discs increased significantly with no loss of height at the last follow-up. ^*∗*^*P* > 0.05 vs. preoperative.

**Figure 4:**
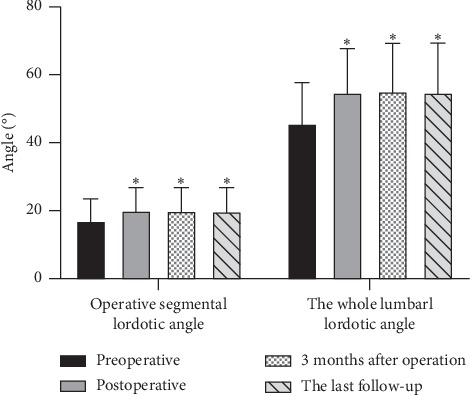
The comparison of sagittal angles. Compared with preoperative, lumbar lordosis angle and segmental lordosis angle increased with no loss at the last follow-up. ^*∗*^*P* > 0.05 vs. preoperative.

The mean follow-up time was 13.5 ± 1.1 months. Operation segments ranged from L3-4 to L4-5. All the patients were with 1-level fusion. The mean operation time was 69.7 ± 8.7 min, and the mean estimated blood loss was 92.6 ± 11.7 ml. The mean postoperative ventral-disc height and dorsal-disc height increased significantly compared with preoperative (*P* < 0.05, Figure 3). A significant postoperative increase was also observed in the mean operative segmental lordotic angle and the whole lumbar lordotic angle (*P* < 0.05, Figure 4).

**Figure 5:**
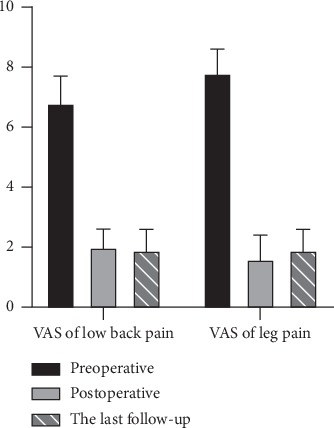
The comparison of the visual analogue scale. Compared with preoperative, the postoperative VAS significantly decreased with no significant increase in the VAS in the last follow-up.

**Figure 6:**
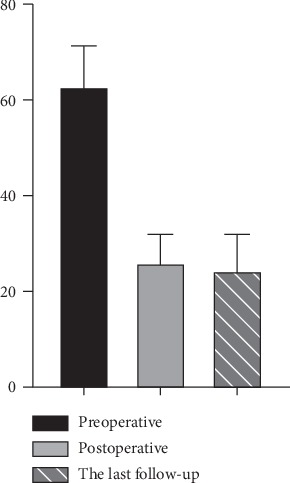
The comparison of ODI. Compared with preoperative, the postoperative ODI significantly decreased. But there existed no statistical difference in ODI reduction at the last follow-up compared with postoperative.

**Figure 7:**
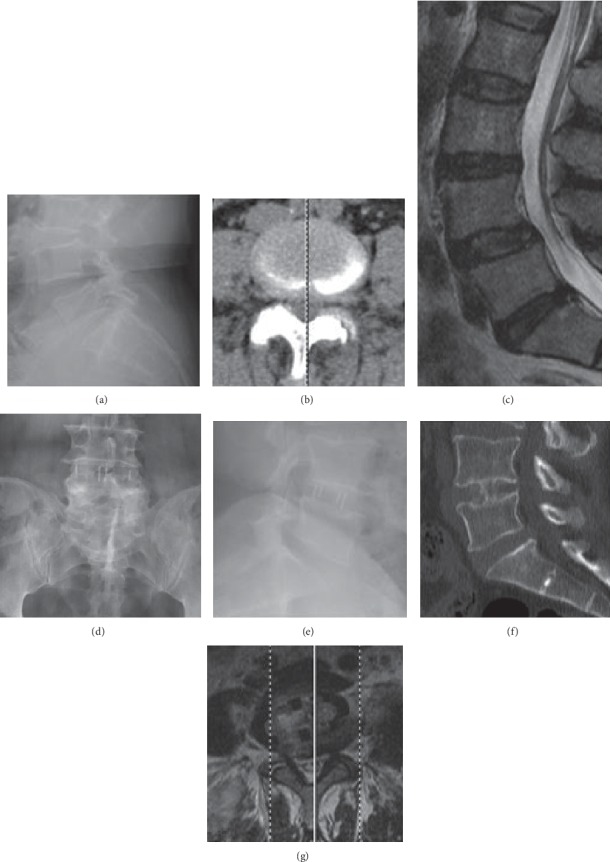
A representative case of OLIF surgery. A patient with degenerative lumbar spondylolisthesis (L4-5, a–c) underwent OLIF surgery (d, e). One year after the operation, fusion and trabeculae were formed with the cage in a desired position (f, g).

The low back pain decreased immediately after OLIF. Compared with preoperative, the postoperative VAS significantly decreased (*P* < 0.05, Figure 5). But there was no significant difference between the last follow-up and the 2 weeks or 3 months. The mean ODI value was significantly improved postoperatively (*P* < 0.05, Figure 6). Postoperative complications included psoas major abscess and intervertebral space infection (1/56). Except for one patient whose cage subsided during the last follow-up, the other patients had good cage position. The one whose cage collapsed complained no symptoms including low back pain (Table 2). Meanwhile, one-year follow-up showed the fusion and no displacement of the cage as we expected (Figure 7).

## 4. Discussion

Low back pain (LBP) is the main complaint of patients who suffered from degenerative lumbar spondylolisthesis [2]. Due to the instability of the lumbar spine, activity often induces severe LBP. OLIF, a minimally invasive technique, indirect decompression, results in disc height restoration and accelerates fusion [3]. Studies have certified that the effective fusion of unstable segments can effectively relieve LBP [4]. In addition, the stand-alone cages have advantages over the traditional posterior cage on account of its size and height [5]. All of the superiority results in reducing the incidence of cage subsidence to relieve postoperative LBP. But the incidence of the subsidence varies among the procedures by using stand-alone cages. Kuang et al. [6] showed that 6% of the cases had cage subsidence with stand-alone cages at 12-month follow-up. Compared with the above studies, the subsidence rate was 2.4% (1/42) with the OLIF procedure. We consider that it is linked to the patient's own condition and the careful operation. Studies have shown that patients with dual energy X-ray absorptiometry (DEXA) *T* scores less than –1.0 who undergo the stand-alone procedure are at a higher risk of cage subsidence [7]. On this account, bone mineral density (BMD) measurements were performed in all patients to meet surgical criteria. Despite meeting the surgical criteria, improper technological performance such as destruction of the endplate can also result in subsidence, which can induce postoperative LBP [8, 9]. In our study, one case presented cage subsidence at the last follow-up with no related symptoms.

Intraoperative infection and postoperative nonfusion can also induce discogenic LBP. Mehren et al. [10] reported that only 0.37% (3/812) of patients had wound infections during the early postoperative period among which had severe postoperative LBP. But Abe et al. [11] showed that 1.9% (3/155) of cases with surgical site infection companied with postoperative LBP. All of the studies above were treated with antibiotics, and the symptoms of back pain were relieved. In our study, one patient (1.8%) complained increased back pain 4 days after surgery. The left psoas major abscess, intervertebral space, and paraspinal infection were found by MRI. The patient's symptoms disappeared after one week of antibiotic infusion treatment. In addition, nonfusion after operation will lead to the pseudoarthrosis and result in discogenic LBP. Several clinical studies have demonstrated that compared with the posterior fusion, stand-alone cages obtained similar fusion rates and even have a high fusion rate (92.3%–98.7%) utilizing these cages without posterior fixation [1, 6, 12]. Our results are consistent with these reports in terms of fusion rates. No cases of nonfusion observed at final follow-up.

Despite some positive reports on the application of stand-alone cages, there were few reports on OLIF technology [9, 12]. Recent research shows that ALIF with stand-alone cages is an effective and safe treatment of degenerative lumbar diseases with less surgical trauma and similar outcomes compared with posterior surgeries [6]. The OLIF procedure implants the cage from the left side of the intervertebral body that leads to satisfactory clinical outcomes. Theoretically, indirect decompression can be achieved by the reduction of the herniation and the elongation of the hypertrophic ligamentum flavum through recovering the intervertebral space. Oliveira et al. reported the LLIF procedure with stand-alone cages to achieve the indirect neural decompression in 21 patients. All radiological parameters significantly improved, including the increase of 41.9% in DH, 24.7% in foraminal area, and 13.5% in foraminal height. Sato et al. [13] reported significant improvements in DH and spinal canal area after the OLIF procedure, and low back pain and leg pain significantly reduced compared with the preoperative. Studies have certified that the reconstruction of LL or lumbar segmental lordosis is essential for the recovery of symptoms and the prevention of adjacent segmental degeneration, even in short segmental surgery [14]. Meanwhile, if there exists serious spondylolisthesis or isthmic spondylolisthesis, OLIF with stand-alone cages is not suitable [15]. It can be combined with posterior fixation, otherwise fusion may fail. Our results showed that all the cases achieved significant improvement in VDH, DDH, and LL with all of the patients meeting the standard. No symptom recurrence was found in all the patients at the last follow-up.

This study still has its limitations. It is a retrospective study without randomized comparison with other lumbar fusion procedures such as PLIF and TLIF. Also, it was a small case series, and the advantages and disadvantages of OLIF with stand-alone cages should be studied using larger series.

## 5. Conclusion

OLIF with stand-alone cages should be considered as a safe and effective option for the treatment of DLS. As a novel technique for spinal surgical management, it showed excellent clinical and radiological outcomes. But careful operation is needed, which can effectively alleviate LBP in patients.

## Figures and Tables

**Table 1:** Main basic demographics and diagnosis of patients.

Characteristics	
Gender (*n*)	Female 24, male 18
Age (yrs)	Mean 58, range 37–78
Fusion levels	L3-4 (4, 9.5%), L4-5 (38, 90.5%)
Follow-up (m)	Mean 13.5, range 12–16

**Table 2:** Radiological and clinical results.

Values	Preoperative	Postoperative	3 months after operation	The last follow-up
Ventral-disc height (mm)	15.5 ± 3.5	17.9 ± 3.1	17.8 ± 3.5	17.8 ± 3.7
Dorsal-disc height (mm)	7.9 ± 1.9	10.3 ± 1.6	10.2 ± 1.8	10.2 ± 1.9
Operative segmental lordotic angle (°)	17.1 ± 6.4	20.1 ± 6.7	20.0 ± 6.8	19.9 ± 6.9
The whole lumbar lordotic angle (°)	45.7 ± 12.0	54.8 ± 12.9	55.2 ± 14.0	54.8 ± 14.5
VAS of LBP	6.8 ± 0.9	2.0 ± 0.6	—	1.9 ± 0.7
VAS of leg pain	7.8 ± 0.8	1.6 ± 0.8	—	1.9 ± 0.7
ODI	62.9 ± 8.4	26.1 ± 5.8	—	24.5 ± 7.4

## Data Availability

The data used to support the findings of this study are included within the article.
